# Guide for Systematization of Care and Nursing Process: educational technology for professional practice

**DOI:** 10.1590/0034-7167-2021-0975

**Published:** 2023-04-14

**Authors:** Susana Lamara Pedras Almeida, Cândida Caniçali Primo, Márcia Valéria de Souza Almeida, Paula de Souza Silva Freitas, Amália de Fátima Lucena, Eliane de Fátima Almeida Lima, Marcos Antônio Gomes Brandão

**Affiliations:** I Universidade Federal do Espírito Santo. Vitória, Espírito Santo, Brazil; II Universidade Federal do Rio Grande do Sul. Porto Alegre, Rio Grande do Sul, Brazil; III Universidade Federal do Rio de Janeiro. Rio de Janeiro, Rio de Janeiro, Brazil

**Keywords:** Nursing Process, Educational Technology, Professional Practice, Nursing Records, Standardized Nursing Terminology, Proceso de Enfermería, Tecnología Educacional, Práctica Profesional, Registros de Enfermería, Terminología de Enfermería Estandarizada, Processo de Enfermagem, Tecnologia Educacional, Prática Profissional, Registros de Enfermagem, Terminologia Padronizada em Enfermagem

## Abstract

**Objective:**

to elaborate and validate the content of a digital guide educational technology on Systematization of Nursing Care and Nursing Process.

**Methods:**

applied research of technological development, developed between 2020 and 2021, in three steps. First, a scoping review was carried out to elaborate the content. In the second step, the content was validated with 46 nurse judges selected for convenience. The minimum criterion of agreement among judges was 80%. The third step consisted of content organization and layout.

**Results:**

the guide content was elaborated from the Federal Nursing Council legislation, scientific articles and textbooks. Content was considered appropriate, relevant and organized by judges.

**Final considerations:**

the digital guide is an alternative that can contribute to the NP execution and implementation, supporting the planning and implementation of actions for quality of care.

## RESUMO

**Objetivo:**

elaborar e validar o conteúdo de uma tecnologia educacional do tipo guia digital sobre Sistematização da Assistência de Enfermagem e Processo de Enfermagem.

**Métodos:**

pesquisa aplicada de desenvolvimento tecnológico, desenvolvida entre 2020 e 2021, em três etapas. Primeiramente, foi realizada uma revisão de escopo, para elaboração do conteúdo. Na segunda etapa, foi validado o conteúdo com 46 juízes enfermeiros selecionados por conveniência. O critério mínimo de concordância entre os juízes foi de 80%. A terceira etapa consistiu na organização e diagramação do conteúdo.

**Resultados:**

o conteúdo do guia foi elaborado a partir das legislações do Conselho Federal de Enfermagem, artigos científicos e livros textos. O conteúdo foi considerado adequado, pertinente e organizado pelos juízes.

**Considerações finais:**

o guia digital é uma alternativa que pode contribuir para a execução e implementação do PE, embasando o planejamento e a implementação de ações para a qualidade da assistência.

## RESUMEN

**Objetivo:**

desarrollar y validar el contenido de una tecnología educativa como guía digital sobre Sistematización de la Atención de Enfermería y del Proceso de Enfermería.

**Métodos:**

investigación aplicada de desarrollo tecnológico, desarrollada entre 2020 y 2021, en tres etapas. En primer lugar, se realizó una revisión de alcance para elaborar el contenido. En la segunda etapa, se validó el contenido con 46 jueces de enfermería seleccionados por conveniencia. El criterio mínimo de acuerdo entre los jueces fue del 80%. La tercera etapa consistió en la organización y maquetación del contenido.

**Resultados:**

el contenido de la guía fue elaborado a partir de la legislación del Consejo Federal de Enfermería, artículos científicos y libros de texto. El contenido fue considerado adecuado, pertinente y organizado por los jueces.

**Consideraciones finales:**

la guía digital es una alternativa que puede contribuir a la ejecución e implementación del PE, apoyando la planificación e implementación de acciones para la calidad de la atención.

## INTRODUCTION

The terms Systematization of Nursing Care (SNC) and Nursing Process (NP) are still treated as synonyms in several Brazilian publications, which contributes to the lack of consensus on these two fundamental concepts for nursing practice, with direct repercussions on the formation of professional identity^(1)^. Moreover, the published literature on SNC brings a diversity of concepts, generating ideological conflicts in the understanding of nursing practice, in the teaching of nursing theories, the nursing process and care methods^(2-3)^.

On the other hand, the Federal Nursing Council (COFEn - *Conselho Federal de Enfermagem*) Resolution 358/2009 points out that the SNC seems to be a broader concept and different from the NP. This resolution states that SNC “organizes professional work in terms of method, staff and instruments, making the operationalization of the NP possible” and defines NP as “a methodological instrument that guides professional nursing care and the documentation of professional practice”^(4)^.

There is a difficulty in using the NP in the daily life of nursing teams as a well-understood and applicable method. Studies indicate that individual and managerial factors can affect the implementation of this method. Among the individual factors, the lack of knowledge of the NP concept stands out. Little knowledge was also strongly linked to nurses’ lack of desire to implement NP in clinical practice. The management factors identified were the scarcity or absence of infrastructure, the incomplete documentation system, the high workload of nursing, the scarcity of human resources, the deficiency of in-service training, among other factors^(5-6)^. Furthermore, the lack of theoretical knowledge, skills and attitudes in relation to the application of NP is also one of the barriers in carrying out NP in the clinical environment^(7)^.

Although nurses recognize the importance of a systematic and deliberate practice, a study carried out in 19 hospitals in Portugal on the NP implementation showed that in practice they still maintain attitudes focused on the care of signs and symptoms^(8)^. Thus, the persistence of practices aimed at the reproduction of routine procedures and traditions reinforces the need to emphasize the adoption of systematized procedures in the care context.

The NP implementation in the United Kingdom was able to solve most care problems, reducing client dissatisfaction with nursing care resulting from the task-oriented approach, with a lack of individualized care and the superficial nature of nurse-patient communication, in addition to nurses’ low level of job satisfaction^(5)^.

In Brazil, a study in 416 sectors of 40 hospitals and clinics in the state of São Paulo, which aimed to identify the prevalence of NP documentation, showed that, in 89.9% of sectors, nurses document only one NP phase, and in 5.8%, they do not do any NP documentation, not even nursing notes. Outpatient clinics, diagnostic support, operating room and obstetric center are the sectors where nurses least documented^(9)^.

For academics, students and nurses directly involved in care, the evidence from the aforementioned studies on documentation problems seems to confirm the challenge presented with the NP implementation. This challenge becomes critical when it is recognized that the NP record, when documented with standardized nursing languages, allows for a better assessment of patients, a more accurate definition of nursing diagnoses and nursing interventions^(10)^.

All these impeding factors and difficulties presented demonstrate the importance of studies that seek to minimize factors that hinder the NP implementation and daily execution^(8)^, since quality of care is directly related to nurses’ attitudes and knowledge when diagnosing, planning and implementing nursing care^(11)^.

Despite the various strategies used by professional bodies and educational institutions, there is a notable gap between the knowledge produced on the concepts of SNC and NP and their applicability in clinical practice. Thus, producing a digital guide is justified as an educational technology to generate new understandings that result in changes in nursing practices and work contexts, in addition to being easy to be made available and consulted in digital format.

## OBJECTIVE

To develop and validate the content of a digital guide educational technology on SNC and NP.

## METHODS

### Ethical aspects

This study was approved by the Research Ethics Committee. Participants received information about the purpose and procedures of the study, and they were asked to sign the Informed Consent Form before data collection.

### Study design

This is applied research of technological development, which followed the criteria established in the Standards for reporting qualitative research: a synthesis of recommendations (SRQR).

### Methodological procedures

This research was carried out between 2020 and 2021, which followed three steps: 1) guide theoretical content elaboration; 2) content validity; and 3) guide layout.

For content elaboration, a scoping review^(12)^ was carried out. Thus, the authors selected as questions for the review: what is SNC? What is NP? What strategies can be used to implement the NP?

We included scientific articles (in English, Portuguese or Spanish and available in full), textbooks and legislation on SNC and NP, nursing staff sizing, nursing theories, concepts and application of NP, nursing instruments and records, without restriction of modality or methodology, published from 2002 to 2021. The initial time frame of 2002 coincides with the publication of regulations that provided, for the first time, in Brazil, on the mandatory implementation and documentation of SNC: COFEn Resolution 272/2002.

The sources consulted were the professional regulation websites, Federal and Regional Councils of Nursing in Brazil and Brazilian states, in addition to bibliographic databases: Latin American and Caribbean Literature in Health Sciences (LILACS); *Base de Dados de Enfermagem* (BDENF); Spanish Bibliographic Index of Health Sciences (IBECS); Medical Literature Analysis and Retrieval System Online (MEDLINE); Cumulative Index to Nursing and Allied (CINAHL); CAPES portal for theses and dissertations; and Cochrane and Scientific Electronic Library Online (SciELO). For electronic search, the tools of the aforementioned databases, libraries and portals were used. A search was also carried out through Google Scholar for material referring to gray literature.

The selected Health Science Descriptors (DeCS) related to NP applicability, concept and teaching were: Nursing Process; Nursing Legislation; Nursing Education, Standardized Nursing Terminology; Nursing Theory; Nursing Diagnosis; Continuing Education, Nursing Records. For search strategy, the Boolean terms AND, OR and NOT were used to compose the search keys to be used for searches in the databases. To complement the search for attributes in the literature, the bibliographic references cited in the selected publications were also used.

Two authors performed a critical reading of the publications, selected to identify and characterize distinctions between the concepts of SNC and NP, identify NP elements that can support its application in nursing practice and verify operational elements of NP and SNC essential to its implementation. This reading included judgment on argument consistency and content reliability of the publications, which were considered, for producing the guide, in line with the regulatory directives contained in COFEn Resolution 358/2009.

Based on the scoping review, a pilot material was organized that was assessed and corrected by four nurses with expertise in NP. After adjustments, we proceed to the second step.

In the second step for content validity, 147 nurses from care, management and teaching areas were selected for convenience. After nurse selection, an invitation letter was sent by e-mail with information about the objective, procedures and instruments to be completed.

Data collection was performed using Google Forms^®^, with a two-section instrument: 1) judge characterization; 2) preliminary version of the guide, still without final layout, for validity of the following items: ability to express the content; organization (order) of content; relevance to the theme; language adequacy; adequacy and clarification of images; organization, sequence and structure.

The authors established as an adequacy criterion the achievement of at least 80% agreement between the judges, and situations where the agreement index was lower than 80% would be reformulated, following the suggestions^(13)^.

For the diagramming step, a team of designers was hired, who worked together with the researchers.

## RESULTS

Based on literature search, the guide content on SNC and NP was prepared in accordance with COFEN legislation provisions on NP. Thus, 47 scientific articles were gathered in the databases, in addition to 24 textbooks on SNC and NP, staff sizing, nursing theories and classifications, concepts and application of NP, instruments for data collection and care documentation.

The digital guide has 45 pages, being composed of three parts: Defining concepts; The Nursing Process; and Strategies for implementing the Nursing Process. The first part covers the topics: What SNC is; Classification methodology; Staff sizing and development; and instruments. The second deals with NP aspects: process steps, registration, use of classifications: International Classification for Nursing Practice (ICNP^®^); NANDA International (NANDA-I); Nursing Outcomes Classification (NOC); and Nursing Interventions Classification (NIC); and the third addresses strategies for implementing the NP. In addition to the textual content, 32 illustrations were prepared, which seek to exemplify, reinforce and self-explain the pertinent content.

Content validity had the participation of 46 (forty-six) nurses from various states of Brazil, most of them from the South and the Southeast, with ages ranging from 27 (twenty-seven) to 69 (sixty-nine years old). Length of professional training varied between 1 (one) and 47 (forty-seven) years. Regarding the degree, 41.3% had a master’s degree, 45.7% a specialization and 13% a doctoral degree.

In the digital guide content validity, in relation to the ability to express the content, experts’ agreement was 94.6%; in item content organization (order), it was 94.4%; in theme relevance, it was 94.6%; in language adequacy, it was 97.2%; in image adequacy and clarification, it was 93.8%; and in organization, sequence and structure, it was 95.8%. The overall agreement index was 95.06%.

In the diagramming step, the written content and figures were reorganized on presentation pages and configured in the format of a digital guide so that it would also meet the needs of those who want to print the material.

The cover and all header and footer layout were prepared by a designer. The insertion of content on the pages resorted to the use of tables that highlight some excerpts that summarize the central ideas of the topics covered. The figures were carefully arranged, highlighting their importance and function of composing the content. The screens were assessed and reformulated by the authors together with the diagrammer.

**Figure 1:**
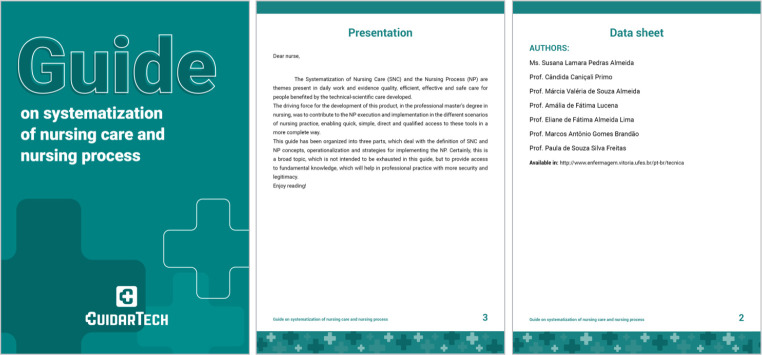
Guide cover, presentation, and summary, Vitória, Espírito Santo, Brazil, 2021

Before starting the didactic content, the guide brings a brief presentation and summary, in which the reader can learn about the content covered and guide their search for a specific topic, as shown in Figure 1.

**Figure 2:**
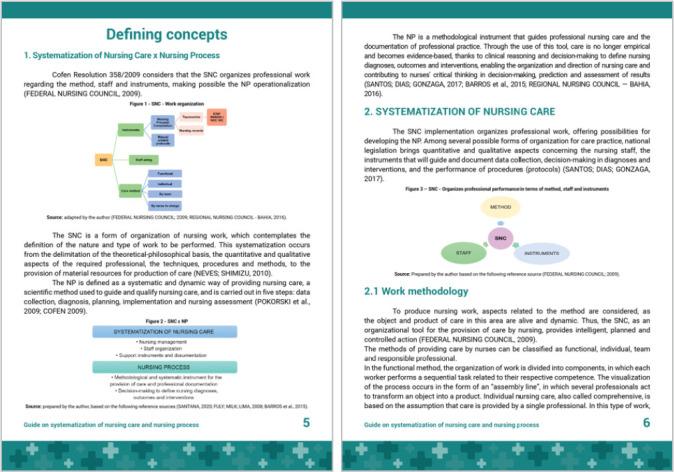
Part 1 of the guide: Defining concepts, Vitória, Espírito Santo, Brazil, 2021

In the first part, the guide presents the distinction between SNC and NP, highlighting the differentiating attributes of the concepts, since understanding the difference between them is an important knowledge for the nursing team, as it is not yet the domain of the entire professional category (Figure 2).

**Figure 3:**
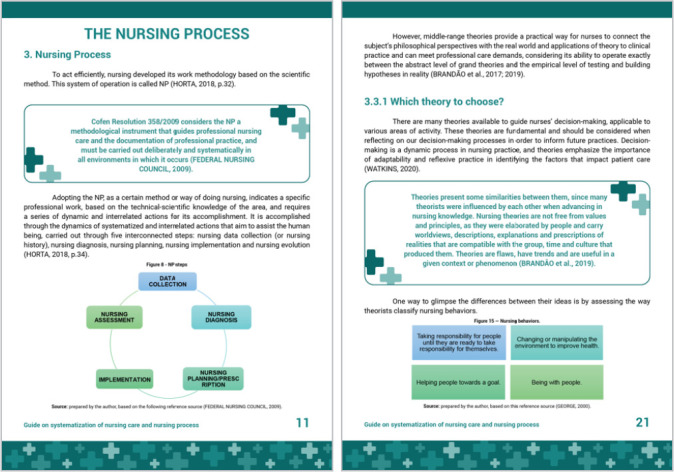
Part 2 of the guide: The Nursing Process, Vitória, Espírito Santo, Brazil, 2021

Part 2 of the guide is entirely dedicated to the NP, addressing the constitutive definition of concept and emphasizing its operational steps. To facilitate understanding, in addition to the definition of each step, figures were also used that represent these dynamics and relationship of the steps, which do not occur in a linear way, but follow a whole reasoning that favors their effectiveness. The legal aspect and the importance of systematizing the nursing consultation were also addressed, bringing its main characteristics clearly and succinctly. The guide exemplifies and presents some of the most well-known and used nursing theories in Brazil (Figure 3).

The guide deals with the importance and legal aspects of nursing records, presenting the conceptual distinction between notes and nursing evolution as well as the mandatory elements for registration. The guide then discusses the standardized nursing language systems used in each NP, addressing the structure and way of applying the NANDA-I, NOC, NIC and ICNP® classifications.

**Figure 4:**
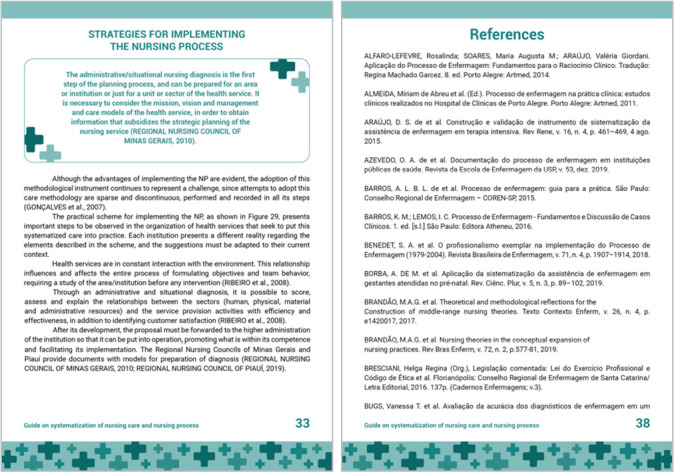
Part 3 of the guide: Strategies for implementing the Nursing Process, Vitória, Espírito Santo, Brazil, 2021

Part 3 of the guide brings two reports on the experiences of hospitals in the SNC and NP implementation, showing some of the strategies and resources available, and then the references used are presented, as shown in Figure 4.

## DISCUSSION

In the first part, the guide presents the distinction between SNC and NP, and it is common to find in practice and in publications that treat NP, care methodology and SNC as synonyms or with different definitions. There is a conceptual conflict that generates difficulty in understanding the professional practice of nursing^(14)^.

The correspondence of meanings attributed to SNC and NP contributes to the lack of consensus on the concept of these two fundamental components for nursing practice, and this has direct repercussions on the formation of professional identity^(1)^.

The second part of the guide addresses NP. The NP implementation showed that nurses recognize the importance of the scientific method to achieve a systematic and deliberate practice, although in practice they still maintain attitudes focused on care focused on the management of signs and symptoms of diseases, to the detriment of the real needs demonstrated by patients^(8)^.

Among the main difficulties in complying with what is recommended in the legislation in relation to NP, the lack of theoretical knowledge, practical exercise and resources, as well as the scarcity of time for its performance, stand out. Nurses’ actions often make doing routine, without sufficient scientific support^(6-7,15)^.

The legal aspect and the importance of Nursing Consultation, at the outpatient level, were also addressed. Nursing consultation should be used as a technology for differentiated care and provided with scientific knowledge that provides understanding and reflection on their health work^(16)^.

NP and nursing consultation should be guided by a theoretical support^(4)^. It is important that nurses know the nursing theories, because their use supports nurses in defining their roles, adequacy and quality of professional performance^(17)^. The guide exemplifies the theme by presenting some of the most known and used theories.

The theoretical support chosen guides data collection, diagnosis definition, action planning and assessment of the results achieved, influencing aspects of nursing record. Practice guided by nursing theory helps to improve quality of care as it allows nurses to articulate what they do for patients and why. Therefore, they should continue guiding their practice through the lens of nursing theory, in addition to assessing the effectiveness of this practice guided by it^(18-19)^.

When performed based on theoretical support and with the use of standardized language, nursing records favor a more effective communication between the health team allowing a continuity of patient care^(20)^.

The guide addresses nursing classifications and records. Standardized language systems are important tools for NP registration, as they qualify documentation and communication between professionals^(21)^. However, incompleteness is still observed in clinical records compromising quality of care and patient safety^(20,22-23)^. On the other hand, the quality of nursing records arouses the interest and need in other professionals to consult them, allowing a better definition of therapy, care and diagnosis, in addition to serving as a basis for assessment and efficiency of quality of care practices^(24)^.

The third part presents the report of two hospitals in the implementation of SNC and NP, showing strategies and resources. The NP implementation favors the strengthening and recognition of nursing through standardization of actions and their records^(5,8,25)^.

The digital guide is an educational technology that contributes to the NP execution and implementation in the different scenarios of nursing practice, enabling quick, simple, direct and qualified access to these tools in a more complete way. Technology can be understood with different meanings, such as technical, scientific knowledge, procedures, processes, products, materials and organizational, educational, information and support systems^(26-28)^.

Nursing technologies are being incorporated into the clinical learning environment as a way of dynamizing the teaching-learning process or in the care and management context, for the NP execution and documentation in order to optimize access to information, records and documentation or to assist decision-making regarding patient care. Technologies also help in the implementation of appropriate practices in the services, organize the work processes, support the execution of health procedures, and update team knowledge^(26-34)^.

The literature is extensive about the development of technologies in nursing. Researches point to the use of qualitative and quantitative approach methodologies, methodological, descriptive, applied technological development, quasi-experimental, cross-sectional, Convergent Care Research, participatory research, action research, integrative or systematic review, User-Centered Design studies, among others^(27-32)^. In this regard, different methods can guide the process of elaboration, validity and application of technologies in teaching, care and management, and the method used in this research has the potential of reproducibility for developing other materials.

Different formats of digital educational technologies are being developed and used in nursing education, such as applications, hypertext, games, videos, virtual environments, virtual learning objects and simulators with virtual reality. In terms of care and management, studies point to the development and use of technologies such as guides, manual/protocol, software/application, teaching material, non-patentable process/technology, diagrams, instructional material, courses, booklets, recommendations, among others^(26-34)^. We emphasized the importance of publicizing the construction/application of technological productions in order to contribute to the advancement of nursing science.

### Study limitations

As a limitation, the technology content validity step is identified, due to the invited judges’ low compliance, pointing out the need for new research in order to expand the number and representativeness of different Brazilian states and practice scenarios of the evaluators.

### Contributions to nursing and health

The digital guide provides nursing professionals with access to material on SNC and NP based on technical-scientific knowledge. In a practical way, themes present in daily work were presented and evidence of quality, efficient, effective and safe care for people. The guide allows access to fundamental knowledge, which will assist in professional practice with more security and legitimacy.

## FINAL CONSIDERATIONS

The “Guide on Systematization of Nursing Care and Nursing Process” is a technological innovation in health, as it encompasses a broad content, based on national and international references on the concepts, implementation strategies and operationalization of SNC and NP. The guide can be important for the entire nursing category, in addition to serving as an instrument that can bring an understanding of NP to professionals from other professional categories, such as managers and multidisciplinary teams, who need to understand the nursing work process for their decision-making.

The guide content was considered by experts as relevant, clear, objective, organized, with adequate and scientifically coherent language and images, and has coherent and relevant organization, sequence and structure. In the global assessment, the agreement rate was 95.06%.

The digital guide is available as a motivating and satisfactory alternative to health education, capable of contributing to the NP execution and implementation, supporting health care planning and implementation of actions aimed at quality of care.

The illustrations help the reader’s understanding of SNC and NP, representing the definition and relationship between these two concepts, and follows a whole reasoning that favors understanding, allowing quick, simple, direct and qualified access to fundamental knowledge, which will help in professional practice based on scientific evidence.

This digital guide is relevant for scientific and technological development, having the potential to generate impact and be applied at local, regional and national levels, as it is available online on the website and can be used in teaching-learning to support nurses and students and provide subsidies for advancing the NP/nursing consultation implementation.
